# Sexual and gender harassment in Swedish workplaces: A prospective cohort study on implications for long-term sickness absence

**DOI:** 10.5271/sjweh.3971

**Published:** 2021-08-31

**Authors:** Katrina Blindow, Fredrik Bondestam, Gun Johansson, Theo Bodin, Hugo Westerlund, Anna Nyberg

**Affiliations:** Institute of Environmental Medicine, Karolinska Institutet, Stockholm, Sweden; Swedish Secretariat for Gender Research, Gothenburg University, Gothenburg, Sweden; Stress Research Institute, Department of Psychology, Stockholm University, Stockholm, Sweden; Department of Public Health and Caring Sciences, Uppsala University, Uppsala, Sweden

**Keywords:** Key terms co-worker, discrimination, gender-based harassment, gender-based violence, sick leave, sexist hostility, sexual harassment, social support, superior

## Abstract

**Objectives:**

This prospective cohort study aimed to investigate gender harassment and sexual harassment as risk factors for prospective long-term sickness absence (LTSA, ≥21 days). Furthermore, support from colleagues was investigated as a moderating factor of this association.

**Methods:**

Information on gender harassment, sexual harassment and support by colleagues were derived from the biannual Swedish Work Environment Survey 1999–2013, a representative sample of the Swedish working population (N=64 297). Information on LTSA as well as demographic and workplace variables were added from register data. Relative rates of LTSA the year following the exposure were determined using modified Poisson regression.

**Results:**

Monthly to daily exposure to gender harassment was a risk factor for prospective LTSA among women [rate ratio (RR) 1.04, 95% confidence interval (CI) 1.02–1.05] and men (RR 1.07, 95% CI 1.04–1.10). Monthly to daily exposure to sexual harassment was also a risk factor for LTSA among women (RR 1.05, 95% CI 1.01–1.10) and men (RR 1.07, 95% CI 1.02–1.13). Exposure to sexual or gender harassment once in the last 12 months was not associated with LTSA. There was no support for an interaction between either of the exposures and support from colleagues in relation to LTSA.

**Conclusions:**

Sexual harassment and gender harassment appear to contribute to a small excess risk for LTSA among women and men. For both kinds of offensive behaviors, the pervasiveness appears to be important for the outcome. The role of support by colleagues was inconclusive and needs further investigation.

In 2019 the International Labour Organization (ILO) adopted the Convention on Combating Violence and Harassment at Work (1) and demanded their members to take active measures to ensure working conditions are free from violence and harassment. The convention particularly emphasizes gender-based violence and harassment (GBVH) with the definition: “harassment and violence directed at persons because of their sex or gender or affecting persons of a particular sex or gender disproportionately and includes sexual harassment” (1). The ILO hereby places GBVH on top of the agenda and provides an inclusive terminology for a spectrum of harmful workplace experiences that have been researched under a variety of concepts.

Determining the prevalence of GBVH is difficult due to methodologic reasons, mainly differences in measurement and underreporting (2–4). In the Swedish Work Environment Surveys quite consistently across 1999–2013, about 18% of women and 6% of men reported some experience of GBHV in the last 12 months, with young women and some occupational groups reporting considerably higher prevalence (5) than others. Most occupational health studies on experiences of GBVH so far focus on sexual harassment as unwanted sexual attention or sexual coercion (6). This way, research reproduces the lay assumption, that it is mainly the sexualizing nature of these acts of sexist hostility that is most harmful (6–8). Sexual harassment is consistently found to go hand in hand with non-sexualizing expressions of sexist hostility, though (9) and some studies suggest, that the pervasiveness of acts of GBVH in the workplace is more decisive for victims’ well-being than the degree of sexualization (6, 10, 11). In consequence, offensive behavior that is based on gender role expectations and norms of heterosexuality without being sexualizing has recently gained more attention under the construct of gender harassment (8, 12, 13). Gender harassment is consistently found to be more prevalent than and to mostly occur without sexualizing offences but has rarely been investigated independently (6, 8, 14, 15). While some studies on sexual harassment include exposed men, to our knowledge, research on gender harassment so far mainly focused on the experiences of women only.

In this study, we base our definition of GBVH on subjects’ acknowledgement of their experiences as harassing. We investigate (i) sexual harassment, experiences that are self-labeled as sexual harassment under the definition of unwanted advances and offensive remarks with a sexual content and (ii) gender harassment, experiences of gender discriminating conduct and sexist remarks about people in general or their suitability for certain work tasks. Besides overt sexist remarks, we include more subtle conduct, such as being ignored, interrupted, or not taken seriously, that the affected person attributes to their gender. Experiencing these more evasive expressions of disrespect has been found to be highly correlated with the experience of open sexist hostility (13, 16).

The harm from experiences of work-related harassment has predominantly been investigated in the transactional stress framework of Lazarus & Folkman (17) insofar as identifying a situation as harassing implies the appraisal of it as being offensive, exceeding one’s resources, and threatening one’s well-being (2, 18). Sexual and gender harassment are similar to other offensive behaviors in communicating a lack of appreciation and respect and can also be understood under the “Stress as Offence to Self” perspective as threatening the self-esteem of the target (19, 20). Berdahl et al (21) point out the stress that GBVH can provoke particularly as it is experienced in the work context, where it threatens the sense of control over essential resources. Some cases of GBVH might also constitute a specific form of workplace bullying, where gender and sexuality are instrumentalized as means of oppression (22).

Studies have found associations of experiences of GBVH with eg, job satisfaction, turnover intention, anxiety, symptoms of depression and PTSD, and even suicide (2, 3, 23, 24). The impact of the psychosocial work environment on the rates and lengths of workers’ sickness absence is widely recognized (25). While workplace bullying could be established as a risk factor for prospective sickness absence (26), the implications of work related GBVH for sickness absence have not been explored sufficiently yet to draw any conclusions (27). A Danish study found an association of unwanted sexual attention with prospective LTSA among men only (22) and a Finnish study, based on women only, found an association of gender discrimination – a term that might measure similar experiences as defined here as gender harassment – with prospective LTSA (28).

Targets of GBVH have to manage their interactions not only with the perpetrator(s) but also with other colleagues, some of whom witness the offensive treatment (29). The quality of the emotional and instrumental social interaction is an important characteristic of workplaces (30) and low social support has been identified as an adverse workplace factor (31). High social support on the other hand can be protective of the negative health effects of interpersonal work-stressors (32) and moderated the association between mobbing and sickness absence in a study by Nielsen et al (33). The perceived social support by colleagues might therefore play a decisive role for the association between experiences of GBVH and LTSA.

The overall aim of the present study was to investigate to what extent experiences of GBVH from superiors and colleagues are associated with LTSA. The study aimed furthermore at investigating whether support by colleagues modifies the association between GBVH and LTSA. The literature suggests considerable gender differences in exposures to and outcomes of GBVH (15, 25–27). Therefore, we studied men and women separately.

## Methods

### Study sample and data

For this prospective cohort study, we pooled data from eight waves (1999–2013) of the Swedish Work Environment Survey (SWES). SWES is a cross-sectional survey, conducted biennially by Statistics Sweden since 1989 on a fairly representative sample of the Swedish working population. The participants are a subsample of the Labor Force Survey (LFS). For the LFS, >20 000 individuals aged 16–74 year are selected by random sampling. After stratification for gender, citizenship, and employment status, a sample representative of the Swedish population is interviewed by phone. Those participants, who are 16–64 in age, gainfully employed and have not been on LTSA or off work for other reasons in the last three months are invited to participate in SWES. The response rate decreased over the study period from 77.59% in 1999 to 56.64% in 2013 (34).

Thirteen participants who reported sexual harassment had missing values in the variable for gender harassment and 10 reporting gender harassment had missing values for sexual harassment. To keep these 23 cases, two different analytical samples were used for the respective exposures. After the removal of individuals with identical personal numbers or missing values in the respective exposure variable, there were 33 349 women and 30 172 men in the study sample for sexual harassment and 33 044 women and 29 989 men in the study sample for gender harassment. Reports on support by colleagues were also taken from SWES. All other data were retrieved from the Swedish Longitudinal Integrated Database for Health Insurance and Labour Market Studies (LISA). LISA contains data on all individuals who are registered in Sweden and ≥16 years and can be connected to SWES through the Swedish personal identification numbers (32). The variables had 0.0–2.1% missing, adding up to just >5% in the analytical models with the most missing values. The Swedish Ethical Review Authority approved the study (No. 2019-05590). SWES participants received written information about the survey, and returning it indicated informed consent.

### Variables

#### Sexual and gender harassment

To measure sexual harassment, the following question was used: “(S)exual harassment refers to unwanted advances or offensive remarks about things that are generally associated with sex.” Gender harassment was measured in direct relation to sexual harassment by the following item: “The next question concerns whether you have experienced conduct (other than that described above) which is based on your gender and that hurts your integrity or is degrading. This can be eg, condescending and ridiculing remarks about men or women in general or in the context of your profession. It can also mean that somebody doesn’t take notice of you or of your contributions because of your gender.” For both items, the participants were asked to rate if they had been subjected to the described conduct by superiors or colleagues on a 7-point Likert-type scale, ranging from “Every day” to “Not at all in the last 12 months”. The variables for sexual harassment and gender harassment were categorized into three values: The first five values were combined to “exposed monthly to weekly” the value “At some time in the last 12 months” was named “exposed once in 12 months”, and rating “Not at all in the last 12 months” was considered as “No exposure in 12 months”.

#### Support by colleagues

Support by colleagues was measured with the question “Are you able to get support and encouragement from colleagues when work feels difficult?” Response alternatives were “Always”, “Mostly”, “Mostly not”, and “Never”. The variable was dichotomized, the first two values were considered as “high support”, the last two as “low support”.

#### Sickness absence

LTSA was defined as having ≥ 21 days of sickness absence, corresponding 7 days of sickness benefits registered by the Swedish Social Insurance Agency (SSIA). In Sweden, the employer pays for the first two weeks of sickness absence, and from day 15, employees can receive sickness benefit from the SSIA. We use net days of sickness absence, one day corresponds either to a full day, the sum of two days with 50%, or four days with 25% of sickness benefit or equivalent payments (eg, for preventive or rehabilitation measures).

### Covariates

Gender was available as the registered status woman or man in the year of survey participation. Age was categorized into five groups: 16–25, 26–35, 36–45, 46–55, 56–64 years. Education was used with the four categories “compulsory”, “2-year upper secondary”, “3 to 4-year upper secondary”, “university < 3 years”, and “university ≥3 years”. Family situation was categorized as single, divorced, separated, or widowed without children; single, divorced, separated, or widowed, no children; married or living with partner, no children; married or living with partner with children. Parental migration background was dichotomized, grouping those with at least one parent born in Sweden and those with both parents born outside Sweden. Country of birth was used with the three categories “Nordic countries”, “other European countries”, and “other continents”. Disposable income was categorized into decentiles. Furthermore, we included a variable for industry classification according to gender composition and main activity, as was first introduced in Cerdas et al (34). The industries were summarized into seven categories: Education (female dominated, working with people); Health and Social Care (female dominated, working with people); Labor intensive services (gender mixed, mixed tasks); Knowledge intensive services (gender mixed, mixed tasks); Public administration (gender mixed, mixed tasks); Goods and Energy Production (male dominated, handling things); Machinery and Operations (male dominated, handling things). LTSA (≥21 days) the year before survey participation was measured like the outcome variable described above and introduced as a covariate or used as an exclusion criterion in some models. In addition, we used a variable for employer exit, that compared the employment status by the end of the year of survey participation with the end of the following year. We dichotomized this variable, grouping all as “employer exit” who left their employer, regardless if they had moved directly to another employer or experienced an episode of unemployment.

### Analytical strategy

The prospective associations between the exposures to gender harassment and sexual harassment on the one hand and LTSA on the other were analyzed with three exposure values (no exposure in 12 months/exposed once in 12 months/exposed monthly to daily), those with no exposure to gender harassment, or respectively to sexual harassment being the reference group. We used Poisson regression analyses with robust error variances (35) to estimate rate ratios (RR) with 95% confidence intervals (CI). Rate ratios measure the relative difference between groups and are here interpreted as differences in risk.

We estimated three models, all of them adjusted for SWES year, age, parental migration background, country of birth, education, family situation, income, and industry classification. We conducted the first model on the full study population. In the second model, we excluded individuals who had LTSA in the year before survey participation (3681 women and 1896 men in the sample for gender harassment and 3709 women and 1904 men in the sample for sexual harassment). We regarded leaving the employer as a potentially competing outcome. Therefore, in the third model we excluded those individuals from analyses who had left the employer by the end of the follow up year (3894 women and 3655 men in the sample for gender harassment and 3923 women and 3689 men in the sample for sexual harassment).

To explore support by colleagues as a potential moderator of the associations between the respective exposures and LTSA, the sample was stratified by low/high support by colleagues. For each group, the prospective association between the exposure to gender harassment and sexual harassment on the one hand and prospective LTSA on the other hand, Poisson regression analysis with robust error variances was performed, including all covariates from the first model and LTSA in the year before survey participation. Additional models, including all the above variables, were conducted to test for interaction on the multiplicative and the additive scale between support by colleagues (high/low) and the exposure to gender harassment or sexual harassment on LTSA, respectively. All statistical analyses were performed in Stata version 16.1 (IBM Corp, Armonk, NY, USA).

## Results

Among women, 11% reported gender harassment and 2% sexual harassment from colleagues or superiors at least once in the last 12 months. Among men, the corresponding prevalence was 4% and 1%. Of those reporting sexual harassment, 64% of the women and 61% of the men also reported gender harassment. As can be seen in table 1, the youngest age group reported most exposure to sexual harassment, and among women, the 26–35-year-olds had the highest exposure to gender harassment. Furthermore, among men, those with a non-Swedish origin were more exposed to sexual and gender harassment than those with a Swedish parent.

**Table 1:** Frequencies and percentages for study variables in all individuals in first column of those individuals who were exposed to sexist hostility in second and those exposed to sexual harassment in third column of women and men respectively. Analytic sample varies between variables due to different number of missing values.

	Women			Men		
	
	All	Gender harassment	Sexual harassment	All	Gender harassment	Sexual harassment
					
	N (%)	N (%)	N (%)	N (%)	N (%)	N (%)
All	33 044/33 349	3678	730	29 989/30 172	1158	287
Sickness absence year after survey						
<21 days in 12 months	29 026 (86.2)	3131 (85.3)	629 (86.3)	28 186 (92.6)	1028 (89.0)	251 (88.9)
≥21 days in 12 months	4630 (13.76)	541 (14.7)	100 (13.72)	2269 (7.45)	127 (11.0)	32 (11.2)
Social support by colleagues						
High	28 998 (87.5)	2874 (78.8)	568 (78.7)	23 900 (79.5)	805 (70.1)	202 (70.9)
Low	4159 (12.5)	775 (21.2)	154 (21.3)	6181 (20.6)	344 (29.9)	83 (29.1)
Age (years)						
16–25	2886 (8.6)	352 (9.6)	132 (18.1)	2366 (7.8)	101 (7.7)	49 (17.1)
26–35	6374 (18.9)	968 (26.3)	275 (37.7)	6285 (20.6)	279 (24.1)	80 (27.9)
36–45	8644 (25.6)	1021 (27.8)	168 (23.0)	7755 (25.4)	296 (25.6)	74 (25.8)
46–55	9374 (27.8)	898 (24.4)	116 (15.9)	8084 (26.5)	284 (24.5)	54 (18.8)
56–64	6434 (19.1)	439 (11.9)	39 (5.3)	6048 (19.8)	198 (17.1)	30 (10.5)
Parental migration background						
One or both parents born in Sweden	30 143 (89.4)	3264 (88.7)	638 (87.4)	27 618 (90.4)	978 (84.5)	243 (84.7)
Parents born outside Sweden	3569 (10.6)	414 (11.3)	92 (12.6)	2919 (9.6)	180 (15.5)	44 (15.3)
Country of birth						
Nordic countries	31 978 (94.9)	3472 (94.4)	684 (93.7)	29 037 (95.1)	1054 (91.1)	263 (91.6)
Other European countries	1004 (3.0)	116 (3.2)	26 (3.6)	855 (2.8)	44 (3.8)	11 (3.8)
Elsewhere	729 (2.2)	90 (2.5)	20 (2.7)	642 (2.1)	59 (5.1)	13 (4.5)
Family situation						
Single/divorced/widowed, no children	8557 (25.4)	1217 (33.1)	331 (45.3)	9506 (31.1)	451 (39.0)	146 (50.9)
Single/divorced/widowed with children	3610 (10.7)	491 (13.4)	91 (12.5)	1403 (4.6)	64 (5.5)	11 (3.8)
Married/living with partner, no children	6554 (19.4)	478 (13.0)	54 (7.4)	5119 (16.8)	151 (13.0)	23 (8.0)
Married/living with partner with children	14 991 (44.5)	1492 (40.6)	254 (34.8)	14 510 (47.5)	492 (42.5)	107 (37.3)
Education						
Compulsory	5796 (17.2)	351 (9.6)	72 (9.9)	5359 (17.6)	170 (14.7)	50 (17.4)
2-year upper secondary	7024 (20.9)	601 (16.4)	107 (14.7)	8284 (27.2)	264 (22.9)	69 (24.0)
3–4-year upper secondary	8051 (23.9)	980 (26.7)	250 (34.3)	8809 (28.9)	335 (29.0)	88 (30.7)
University <3 years	5002 (14.9)	583 (15.9)	103 (14.1)	3127 (10.3)	157 (13.6)	35 (12.2)
University ≥3 years	7815 (23.2)	1161 (31.6)	198 (27.1)	4927 (16.2)	229 (19.8)	45 (15.7)
Disposable income (SEK)						
107 700	3979 (11.8)	341 (9.3)	88 (12.1)	2450 (8.0)	95 (8.2)	33 (11.5)
107 800–132 500	4237 (12.6)	397 (10.8)	80 (11.0)	2166 (7.1)	92 (7.9)	27 (9.4)
132 600–151 300	3960 (11.8)	360 (9.8)	93 (12.7)	2499 (8.1)	116 (10.0)	30 (10.5)
151 400–168 400	3658 (10.9)	360 (9.8)	76 (10.4)	2739 (9.0)	94 (8.1)	22 (7.7)
168 500–186 700	3491 (10.3)	411 (11.2)	74 (10.1)	2939 (9.6)	115 (9.9)	32 (11.2)
186 800–207 700	3436 (10.2)	373 (10.1)	70 (9.6)	3015 (9.9)	112 (9.7)	38 (13.2)
207 800–232 200	3270 (9.7)	399 (10.9)	68 (9.3)	3131 (10.3)	131 (11.3)	30 (10.5)
232 300–265 000	3020 (9.0)	364 (9.9)	71 (9.7)	3406 (11.2)	130 (11.2)	24 (8.4)
265 100–324 100	2579 (7.7)	367 (10.0)	61 (4.9)	3848 (12.6)	151 (13.0)	24 (8.4)
> 324 200	2082 (6.2)	306 (8.3)	49 (6.7)	4345 (14.2)	122 (10.5)	27 (9.4)
Industry classification						
Education	5613 (17.0)	491 (13.5)	93 (12.9)	1834 (6.1)	113 (9.9)	16 (5.7)
Health and social care	10 443 (31.5)	766 (21.0)	93 (12.9)	1602 (5.4)	168 (14.7)	40 (14.1)
Labor intensive services	7046 (21.3)	810 (22.2)	207 (28.6)	6438 (21.5)	262 (33.9)	70 (24.7)
Knowledge intensive services	3569 (10.8)	542 (14.9)	112 (15.5)	4336 (14.5)	130 (11.4)	38 (13.4)
Public administration	2378 (7.2)	352 (14.9)	62 (8.6)	1882 (6.3)	86 (7.5)	13 (4.6)
Goods and energy production	2764 (8.4)	475 (13.0)	103 (14.3)	7655 (25.5)	220 (19.3)	66 (23.3)
Machinery operations	1308 (4.0)	210 (5.8)	53 (7.3)	6223 (20.8)	163 (14.3)	40 (14.1)
Employer exit						
No employer exit	28 392 (86.0)	2987 (82.2)	557 (77.5)	25733 (86.1)	969 (85.7)	234 (82.1)
Employer exit	4641 (14.1)	646 (17.8)	162 (22.5)	4151 (13.9)	162 (14.3)	51 (18.0)
Sickness absence year before survey						
<21 days in 12 months	29 833 (88.6)	3263 (88.7)	653 (89.5)	28 525 (93.5)	10 661 (91.6)	256 (89.5)
≥21 days in 12 months	3847 (11.4)	414 (11.3)	77 (10.5)	1987 (6.5)	97 (8.3)	30 (10.5)

Elevated risks of LTSA (≥21 days) were found among women who experienced gender harassment (RR 1.04, 95% CI 1.02–1.05) or sexual harassment (RR 1.05, 95% CI 1.01–1.10) from colleagues or superiors monthly to daily in comparison to those who did not report the respective exposure (table 2). An elevated risk of LTSA (≥21 days) was also found among men who reported monthly to daily gender harassment (RR 1.07, 95% CI 1.04–1.10) or sexual harassment (RR 1.07, 95% CI 1.02–1.13) from colleagues or superiors (table 2).

**Table 2:** Long-term sickness absence (≥21 days) for three exposure categories of gender harassment and sexual harassment, using no exposure as the reference. Relative rates based on Poisson regression analyses with robust standard errors. Adjusted for survey wave, age, parental migration background, country of birth, education, family situation, income and industry classification.

Exposure	Cases in respective models	Full sample	Individuals with LTSA year before excluded	Individuals with workplace exit excluded
			
	N	RR (95% CI)	RR (95% CI)	RR (95% CI)
Women				
Gender harassment				
Not in 12 months	28 746/25 472/21 815	1	1	1
0nce in 12 months	2339/2089/1728	1.02 (1.00–1.03)	1.02 (1.00–1.03)	1.02 (1.00–1.03)
Monthly to daily	1298/1141/900	1.04 (1.02–1.05)	1.04 (1.02–1.05)	1.04 (1.02–1.06)
Sexual harassment				
Not in 12 months	31 958/28 325/24 183	1	1	1
0nce in 12 months	495/439/348	1.00 (0.98–1.03)	0.99 (0.97–1.02)	0.99 (0.96–1.02)
Monthly to daily	227/207/148	1.05 (1.01–1.10)	1.06 (1.01–1.10)	1.07 (1.02–1.13)
Men				
Gender harassment				
Not in 12 months	28 177/26 376/22 601	1	1	1
0nce in 12 months	734/675/586	1.02 (1.00–1.04)	1.02 (1.00–1.04)	1.03 (1.01–1.05)
Monthly to daily	402/366/296	1.07 (1.04–1.10)	1.05 (1.02–1.08)	1.06 (1.03–1.10)
Sexual harassment				
Not in 12 months	29 210/27 336/23 409	1	1	1
0nce in 12 months	159/140/116	1.01 (0.97–1.05)	1.01 (0.97–1.05)	1.02 (0.98–1.06)
Monthly to daily	123/112/92	1.07 (1.02–1.13)	1.05 (0.99–1.10)	1.07 (1.01–1.14)

Among women and men, one time and monthly-to-daily exposure to gender harassment and monthly-to-daily exposure to sexual harassment were associated with LTSA the year before survey participation, adjusted for covariates. In models where those who had LTSA the year before the survey were excluded, the associations between the respective exposures and prospective LTSA remained statistically significant among women but not men. We considered leaving the employer (either into unemployment or other employment) a competing outcome to LTSA. A high number of exposed men and women had left their employer by the end of the year following survey participation. However, results from models in which they were excluded were similar to those in which they were included, except for exposure to gender harassment once in 12 months among men, which was statistically significant in the former (RR 1.03, 95% CI 1.01–1.05) but not the latter.

Table 3 displays the results from the fully adjusted analysis stratified by high/low support from colleagues. Excess risks of LTSA were found among women with high but not those with low support from their colleagues who also reported monthly-to-daily exposure to gender harassment (RR 1.03, 95% CI 1.01–1.05) or sexual harassment (RR 1.06, 95% CI 1.01–1.11). Men who reported monthly-to-daily experiences of gender harassment had an elevated risk of LTSA in both strata of support from colleagues. In the group with low support from colleagues, also men exposed to gender harassment once in 12 months had an elevated risk of LTSA (RR 1.05, 95% CI 1.01–1.09). For sexual harassment, the association was only statistically significant in the group with low support from colleagues (RR 1.13, 95% CI 1.02–1.24).

**Table 3:** Associations of the exposure to gender harassment and sexual harassment and long-term sickness absence (≥21 days) in the following year, stratified by low or high social support by colleagues. Relative rates based on Poisson regression analyses with robust standard errors. Adjusted for survey wave, age, migration background, country of birth, education, family situation, income, industry classification and long-term sickness absence (≥21 days) in the year before survey participation.

Exposure	High support		Low support	
	
	N	RR (95% CI)	N	RR (95% CI)
Women				
Gender harassment				
Not in 12 months	25 214	1	3155	1
0nce in 12 months	1910	1.01 (1.00–1.02)	415	1.03 (0.99–1.06)
Monthly to daily	936	1.03 (1.01–1.05)	347	1.03 (0.99–1.06)
Sexual harassment				
Not in 12 months	27 764	1	3797	1
0nce in 12 months	393	1.00 (0.97–1.02)	99	1.00 (0.94–1.06)
Monthly to daily	167	1.06 (1.01–1.11)	55	0.98 (0.91–1.06)
Men				
Gender harassment				
Not in 12 months	22 355	1	5513	1
0nce in 12 months	536	1.01 (0.98–1.03)	193	1.05 (1.01–1.09)
Monthly to daily	256	1.05 (1.02–1.09)	142	1.08 (1.03–1.13)
Sexual harassment				
Not in 12 months	23 088	1	5804	1
0nce in 12 months	116	1.02 (0.97–1.07)	43	0.95 (0.90–1.00)
Monthly to daily	81	1.04 (0.98–1.10)	40	1.13 (1.02–1.24)

Low support from colleagues was a risk factor for LTSA among women only (RR 1.03, 95% CI 1.02–1.04). The analysis for interaction between support from colleagues (high/low) and gender harassment or sexual harassment, respectively, on prospective LTSA showed no statistically significant interaction on the multiplicative or additive scales (P-values>0.05).

## Discussion

In this prospective cohort study, we found a small excess risk of LTSA (≥21 days) among Swedish women and men who experienced gender or sexual harassment by a superior or colleague on a daily to monthly basis. In the analyses stratified for support from colleagues, this association was statistically significant only among women with high support and more pronounced among men with low support.

A Finnish study conducted on women only found an association of gender discrimination and LTSA (≥11 days) in the subsequent three years (28). Gender discrimination was not defined in this study, so it is unclear which experiences participants self-identified. Our study confirms the findings of this study and adds by assessing more specific exposures, particularly differentiating between sexualizing and non-sexualizing experiences. Our findings are also in line with Nabe-Nielsen et al (36), who found an association between unwanted sexual attention and LTSA (≥31 days) in the subsequent two years. Clausen et al (19) on the other hand found no association between unwanted sexual attention and LTSA (≥8 weeks) in the following year among women working in elderly-care services. These three studies did not differentiate the source of harassment though, and a great share of the reported harassment is most likely attributable to others than colleagues or superiors, such as patients or customers (2, 37), which makes them less comparable to the present study. Hogh et al (22) investigated unwanted sexual attention specifically from co-workers (of any status position) and found an association with LTSA (≥3 weeks) in the 18 months follow-up only among men. To explain the gender difference in their results, Hogh et al suggest that men might be less inclined to self-label unwanted sexual attention and therefore may have reported only more severe cases. Our study contributes with a different explanation for gender differences in studies such as Hogh et al’s, that rely on binary exposure-variables. Rather than severity, it might be pervasiveness of the exposure that differs between women and men reporting harassment. In our study sample, the proportion reporting highly frequent exposure (daily to weekly) was considerably higher among the exposed men than the exposed women. This might be due to gender differences in reporting, as some studies suggest (12, 30), or less frequent exposure could be more common among women than men. Either way, our results emphasize the importance of taking pervasiveness of the exposure into account.

Our analysis gave rather small effect sizes. The above mentioned studies with longer follow-up time found more pronounced associations between the respective exposure and LTSA (22, 28, 37), while the study that also measured LTSA with one year follow-up found no association (19). This might point to a slower development of health conditions that give grounds for LTSA. This could be due to a deterioration of the work situation or GBVH exhausts the exposed over time.

There was no support for an interaction between GBVH and support, yet the results from the stratified analysis give grounds to some thoughts. From the perspective of high support from colleagues as a buffer against interpersonal stressors (32), the analysis stratified for high/low support gave an unexpected result among women. The association of GBVH with LTSA was only found among women with high perceived support. women who perceive their colleagues as supportive and report GBVH might have experienced the mistreatment to a higher extent from a superior, and GBVH is consistently found to be perceived as more threatening and have a higher impact on targets when it comes from a higher-level employee (6). Among men, the results are more supportive of the assumption that high support from colleagues plays a protective role through several mechanisms. The transactional model of stress and coping of Lazarus & Folkman (17) considers stress a reaction to adverse experiences that exceed available coping resources. Social support from colleagues can be a resource of practical and emotional value, as supportive colleagues can provide information, practical assistance, and appreciation that other colleagues or superiors withhold. However, as no interaction was found, we cannot confirm the hypothesis of social support as a buffer in the relation between experiences of GBVH and prospective LTSA.

### Strengths and limitations

Compared to other studies on work related GBVH, where the majority are based on one employment sector and a small sample, this study has high external validity, as the study population was fairly representative of the working population in Sweden. This being said, the decreasing response rate (56% in 2013) and lower survey participation of young people and those with low education, income or a foreign background still provide for some bias (38). A limitation in this study is the use of single item questions for the exposures, that entirely rely on the self-identification of the respondents. GBVH is consistently found to be highly underreported and only a part of those experiences that are defined by researchers as GBVH are acknowledged as such by respondents. In general, more reliable exposure assessments are crucial to move the research field of GBVH forward. A strength of the present study was, however, the possibility to differentiate the frequency of the exposures. Considering the significant differences found in earlier studies, information on the job positions of targets and perpetrators could have been of much informative value. The prospective design with different sources of measurement, ie, self-reports for the exposures and register-based data for all other information, contributes to good internal validity. This way common method variance could be reduced. Though the measure of sickness absence based on register data generally is an advantage, it comes with weaknesses. Not everyone who is gainfully employed qualifies for sickness benefits from the SSIA. Employees with precarious working conditions, such as short-term contracts or work on demand can face difficulties accessing sickness benefits. As they have been found to be at a particular risk of GBVH (39), these circumstances may have led to an underestimation of the association between the exposures and LTSA. Another limitation is, that we only investigated sickness absence of ≥21 days. Targets of GBVH might have a higher number of short spells of sick leave rather than one long spell. Also, it is possible, that employees with poorer health were more exposed or more inclined to report exposure, which could have led to an overestimation of the association with LTSA. However, we performed analyses excluding those with LTSA in the year before survey participation and found mostly stable results.

### Concluding remarks

The findings suggest the vulnerability of both women and men to gender harassment and sexual harassment in the workplace, particularly when they occur more frequently. The effect sizes were small, however, they proved very robust. The study does not support the commonsense perception that sexualizing conduct is more problematic for the exposed than non-sexualizing experiences of sexist hostility. Rather the pervasiveness of the exposure (here operationalized as exposure-frequency) seems to be decisive, and the results give support to the position that gender harassment must be taken as seriously as sexual harassment. Our findings on the role of support by colleagues were inconclusive. Future studies should explore more potential moderators to further our understanding of the dynamics of GBHV in the workplace. Urgently needed are studies that differentiate the job position of those reporting exposure and the perpetrators as well as the pervasiveness of the experienced GBVH.

### Declaration of interests

The authors declare no conflict of interests.
